# Identifying the white matter pathways involved in multiple sclerosis-related tremor using diffusion tensor imaging

**DOI:** 10.1177/20552173231208271

**Published:** 2023-11-08

**Authors:** Ahmed Bayoumi, Khader M. Hasan, Jorge Patino, Zafer Keser, Joseph A. Thomas, Refaat E. Gabr, Claudia Pedroza, Arash Kamali, Mya C. Schiess, Jerry S. Wolinsky, John A. Lincoln

**Affiliations:** Department of Neurology, 12339 McGovern Medical School at UTHealth, Houston, TX, USA; Department of Radiology, 12339 McGovern Medical School at UTHealth, Houston, TX, USA; Department of Neurology, 12339 McGovern Medical School at UTHealth, Houston, TX, USA; Department of Neurology, 6915 Mayo Clinic, Rochester, MN, USA; Department of Neurology, 12339 McGovern Medical School at UTHealth, Houston, TX, USA; Department of Radiology, 12339 McGovern Medical School at UTHealth, Houston, TX, USA; Department of Pediatrics, McGovern Medical School at UTHealth, Houston, TX, USA; Department of Radiology, 12339 McGovern Medical School at UTHealth, Houston, TX, USA; Department of Neurology, 12339 McGovern Medical School at UTHealth, Houston, TX, USA

**Keywords:** Multiple sclerosis, tremor, diffusion imaging, white matter pathways, tractography, proprioception

## Abstract

**Background:**

Tremor affects up to 45% of patients with Multiple Sclerosis (PwMS). Current understanding is based on insights from other neurological disorders, thus, not fully addressing the distinctive aspects of MS pathology.

**Objective:**

To characterize the brain white matter (WM) correlates of MS-related tremor using diffusion tensor imaging (DTI).

**Methods:**

In a prospective case-control study, PwMS with tremor were assessed for tremor severity and underwent MRI scans including DTI. PwMS without tremor served as matched controls. After tract selection and segmentation, the resulting diffusivity measures were used to calculate group differences and correlations with tremor severity.

**Results:**

This study included 72 PwMS. The tremor group (n = 36) exhibited significant changes in several pathways, notably in the right inferior longitudinal fasciculus (Cohen's *d *= 1.53, *q* < 0.001) and left corticospinal tract (*d* = 1.32, *q* < 0.001), compared to controls (n = 36). Furthermore, specific tracts showed a significant correlation with tremor severity, notably in the left medial lemniscus (Spearman's coefficient [*r_s_p*] = −0.56, *p* < 0.001), and forceps minor of corpus callosum (*r_s_p* = -0.45, *p* < 0.01).

**Conclusion:**

MS-related tremor is associated with widespread diffusivity changes in WM pathways and its severity correlates with commissural and sensory projection pathways, which suggests a role for proprioception or involvement of the dentato-rubro-olivary circuit.

## Introduction

Tremor has been historically recognized as one of the clinical manifestations of MS,^1^ with an estimated prevalence of up to 45%,^2^ though this may be an underestimation given the transient nature of the symptoms. Tremor is among the strongest predictors of impaired quality of life in patients with multiple sclerosis (PwMS),^3^ and can impact activities of daily living (ADLs).^2^ Current treatment modalities for MS-related tremor are lacking in their justification, measured efficacy, or outcomes.^4^ Deep brain stimulation is one of the few available options for severe medication-resistant tremor, though, it shows limited efficacy in treating MS-related tremor compared to essential tremor or Parkinson's disease-related tremor^5^ while causing permanent adverse effects in up to 25% of the cases.^6^ One reason may be that MS differs fundamentally from other causes of tremor with variable topography of pathology in the brain and spinal cord. Many studies on MS-related tremor are conducted with prior assumptions of involved neurocircuitry drawn from other causes of tremor, often scrutinizing cerebello-thalamocortical networks.^4,7,8^

Diffusion tensor imaging (DTI) is a reliable, validated technique that quantitatively assesses random water mobility, providing information about the tissue microstructure integrity measured in terms of diffusivity.^9^ In a medium without an oriented microstructure, diffusivity is similar in three axial directions. Mean diffusivity (MD) is the average diffusivity in the three directions and can be used to describe the structural integrity of the tissue. The directional diffusivities, radial diffusivity (RD), and axial diffusivity (AD) describe water movement in compact white matter tracts. It has been previously shown that increased RD is associated with myelin injury,^10^ while AD correlates with axonal injury.^11^ Fractional anisotropy (FA) is a scalar value calculated from the directional diffusivities and reflects the directionality of diffusion. FA has been validated as a measure of myelination status.^12^ Damage to white matter (WM) causes an increase in mean diffusivity and a decrease in FA due to a loss of directional diffusion.^13^

In this study, we aimed to characterize the WM microstructural changes associated with MS-related tremor as well as describe how these changes correlate with tremor severity using diffusion tensor imaging (DTI), to offer a better understanding of MS-related tremor taking into consideration the distinct nature of the disease.

## Methods

### Subjects

In this prospective case-control study, PwMS diagnosed using 2017 McDonald's criteria,^14^ having limb-predominant MS-related tremor not attributable to another etiology and British Medical Research Council Rating Scale motor strength in all extremities ≥ 4 were recruited, between 2019 and 2021, from the comprehensive MS care clinic at UT Physicians, McGovern Medical School, UTHealth. Subjects were sequentially enrolled, and subsequent adjustments to recruitment were made based on gender, age, and tremor severity to form the tremor group (MS-T). This method allowed us to minimize imbalance in our sample based on demographic and clinical characteristics. An additional group of PwMS matched by age and gender, without a clinically evident tremor, provided a comparison control group (MS-C) with a ratio between cases and controls of 1:1. The study was approved by the Institutional Review Board for the University of Texas Health Science Center at Houston – UTHealth (IRB HSC-MS-19-0305). All subjects participated in the study after giving written informed consent following approved procedures by the Committee for the Protection of Human Subjects of McGovern Medical School, UTHealth.

### Clinical evaluation

Symptomatic medications to lessen tremor severity, if present, were held for at least 24 h prior to clinical assessments only if medically cleared by the treating physician and with additional informed consent from the patient. Currently, there is no consensus as to rating scales to evaluate MS-related tremor severity. We used The Essential Tremor Rating Assessment Scale (TETRAS), a previously validated measure used in essential tremor that correlates strongly with other measures previously usurped to assess MS-related tremor.^15,16^ TETRAS quantifies tremor severity and its impact on ADLs with a focus on upper extremity action tremor while also assessing head, face, voice, and lower limbs. Evidence indicates that TETRAS offers improved characterization of tremor, especially in instances of severe tremor.^16^ As an additional method of evaluating tremor, Archimedean spiral drawings were obtained and scored according to the Tremor Research Group instructions.^17^ Upper limb ataxia, which has a possible confounding effect due to overlap in etiology and neuroanatomical correlates with tremor, was scored using the Scale for Assessment and Rating of Ataxia (SARA).^18^ Lastly, limb spasticity and extremity motor strength were assessed using the Modified Ashworth Scale and the British Medical Research Council Rating Scale, respectively. Disability data as quantified by the expanded disability severity scale (EDSS) was assessed as previously described.^19^ All clinical assessments were performed by raters not involved in image analysis.

### Image acquisition

MRI was performed using a 3T Philips Ingenia research scanner (Philips Medical Systems, Best, Netherlands). High-resolution 3D T1-weighted, used for anatomical registration, were acquired at the start of each scanning session, as well as T2-weighted fluid-attenuated inversion recovery (FLAIR) images. Diffusion-tensor images (DTI) were obtained using a single-shot spin-echo diffusion-sensitized echo-planar imaging sequence with a balanced Icosa21 tensor encoding scheme.^20^ DTI quality control throughout data acquisition was assured and post-processed as detailed previously.^21^ Technical details on acquisition hardware and sequences can be found in the supplementary.

### Image analyses

**Figure 1.**
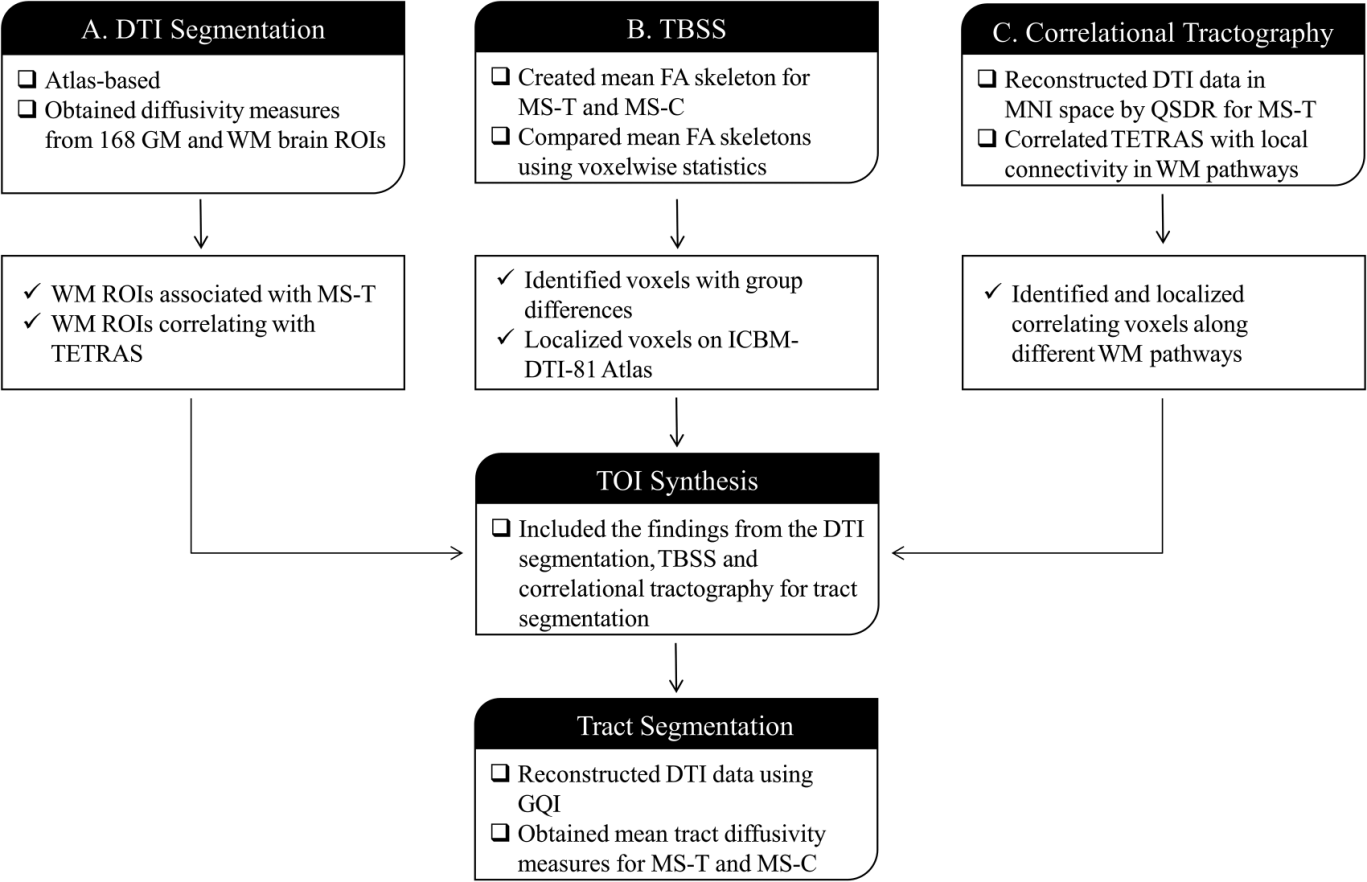
**Image analysis flow.** **(A)** DTI Segmentation. **(B)** TBSS. **(C)** Correlational tractography. GM, grey matter; WM, white matter; ROIs, regions-of-interest; MNI, montreal neurological institute.

White matter lesions were segmented on the T2-FLAIR and T1-weighted images using BIANCA,^22^ and validated by two trained raters. Voxel-wise lesion probability maps were created as previously described.^23^ A two-step approach was employed to identify and examine the potentially associated WM fiber pathways in an agnostic manner, summarized in Figure 1. The first step, tract selection, aimed to synthesize the tracts-of-interest (TOIs) using three independent techniques scoping different anatomical resolutions. The second step, tract segmentation, aimed to individually segment the TOIs for each subject. Further details on the image analyses experiments setup can be found in the supplementary.

#### Tract selection

- Atlas-based DTI segmentation: in this step, regional diffusivity values were obtained for various brain regions using an atlas-based method. By comparing the two groups, we identified WM regions potentially associated with MS-T.
- Tract-Based Spatial Statistics (TBSS): in FMRIB Software Library, TBSS^24^ was used to create a mean FA image and skeleton for each group by averaging their FA images in a standard space and *voxelwise* comparisons were made to identify any significant differences. Using *atlasquery*, the identified voxels were localized on the ICBM-DTI-81 white-matter labels atlas.
- Correlational tractography: this technique uses connectometry, a more sensitive method compared to region-of-interest approaches,^25^ to statistically infer the association with the study variable within WM pathways by analyzing the local voxel-wise connectomes. In MS-T, diffusion data were reconstructed in standard space. This analysis was conducted using quantitative anisotropy (QA)^26^ and FA separately as a tracking index and TETRAS as the study variable while controlling for the effects of age and gender.

#### Tract segmentation

Tractography offers a method for WM segmentation that can effectively accommodate individual anatomical variations in tract dimensions and shapes. A recent study highlighted its feasibility in MS, as it does not introduce systematic differences when tracking in normal appearing WM, T2 lesions or T1 lesions.^27^ For each subject, 53 pathways were tracked using DSI Studio and a supervised automated method with a deterministic tractography algorithm^26^ with augmented strategies to improve reproducibility.^28^ The diffusion data were reconstructed using generalized q-sampling imaging (GQI)^29^ with a diffusion sampling length ratio of 2. GQI-based reconstruction has been validated on a similar acquisition protocol and has been shown to more consistently and accurately resolve crossing fibers compared to DTI-based reconstruction.^30^ The mean tract FA, MD, RD, and AD were obtained from the segmented pathways for MS-T and MS-C.

### Statistical analysis

Statistical analyses for group differences were conducted using *jamovi* (version 2.3), with a significance level set at *p* < 0.05. Demographic data's standard deviation was estimated using student's t-test, while the EDSS scores were compared using the Mann-Whitney U-test after testing for normality. Categorical variables were analyzed using contingency tables and chi-squared tests. To compare the variance in mean diffusivity measures of the pathways between MS-T and MS-C, ANCOVA was employed while controlling for the effects of age and gender, following residual plotting and inspection. Mean difference and effect size, quantified by Cohen's d (*d*), are reported. The p-values were adjusted for false discovery rate (*q*) using the Benjamini-Hochberg procedure.

In R (version 4.1.1), we studied the correlation between tract diffusivity measures and tremor severity in MS-T. Mean tract FA, MD, RD, and AD served as independent variables in the LASSO-partial ridge (LPR) regression.^31^ The tremor severity measures (TETRAS and its left, TETRAS-L, and right component, TETRAS-R, as well as left and right Archimedean spiral scores, ARCH-L and ARCH-R, respectively) were considered as dependent variables. LPR regression was performed using the HDCI package with 1000 paired bootstraps. The lambda value was selected using 10-fold cross-validation to achieve the most regularized model within one standard error of the minimum (lambda.1se), minimizing the mean squared error. This analysis also included age, gender, and ataxia severity (SARA) as non-penalized explanatory variables. The partial correlation coefficients (*r_s_p*) were calculated using Spearman's rank correlation for the selected pathways and their corresponding tremor measures while controlling for the effects of age, gender, and ataxia severity.

Raw data was generated at UTHealth. Anonymized derived data may be shared upon reasonable request to the corresponding or senior authors with researchers who provide a methodologically sound non-commercial proposal, subject to restrictions according to participant consent and data protection legislation.

## Results

### Demographic and clinical data

**Figure 2.**
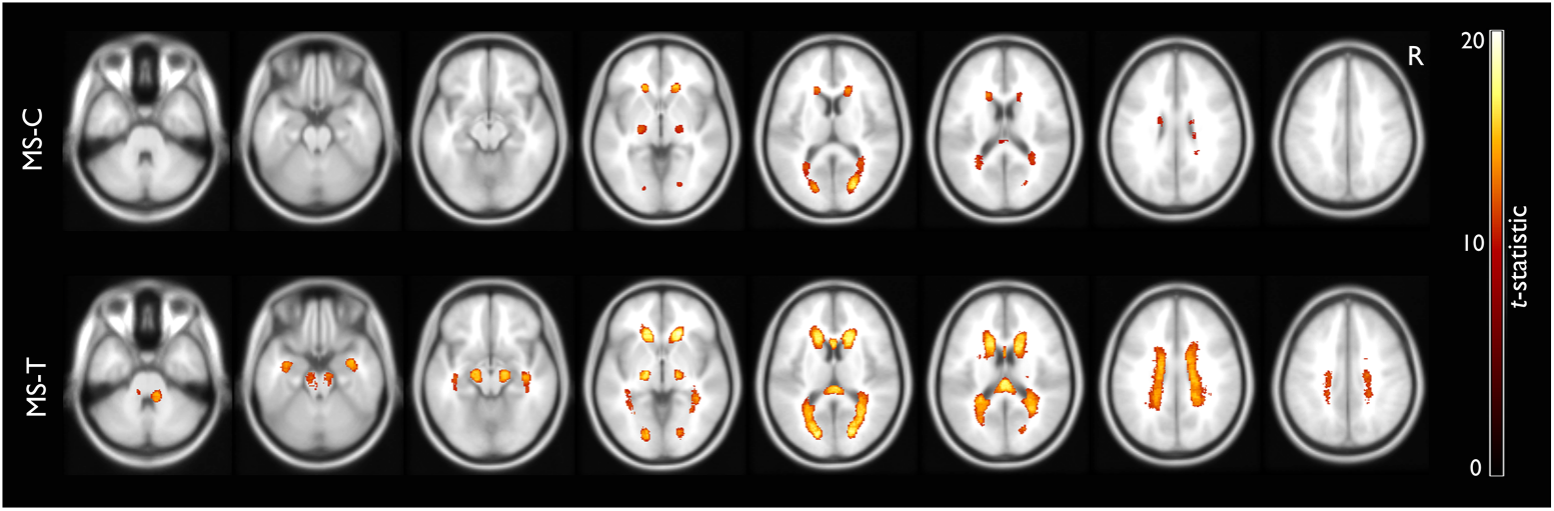
**Lesion probability maps.** Depicting the average lesion burden distribution in MS-C and MS-T, *t*-statistic at *p* < 0.05 calculated from permutation tests to adjust for multiple comparisons.

A total of 72 PwMS were included in this study, Table 1 summarizes their demographic and clinical data. MS-T had higher total EDSS scores, particularly in ambulation and cerebellar functions compared to MS-C, and a higher lesion burden visually more prominent in the brain stem, the periventricular areas, splenium, and genu of corpus callosum. Lesion probability maps for both groups can be seen in Figure 2.

**Table 1.** Demographic and clinical measures.

	MS-C (n = 36)	MS-T (n = 36)	*p*-value
**F : M**	21: 15	21: 15	
**Age ± SD (years)**	47.5 ± 12.7	46.7 ± 11.5	0.8
**Disease duration ± SD (years)**	10.5 ± 9	12.7 ± 6.6	0.2
**MS subtype**			0.5
RRMS	27	23	
PPMS	4	4	
SPMS	5	9	
**DMT**			0.2
Injectable^a^	5	5	
Oral^b^	13	5	
Infusion^c^	14	21	
None	4	5	
			
EDSS total*	2.3	4.7	
	2 (2.5)	6 (4.5)	< 0.001
Visual functions*	0.4	0.2	
	0 (0)	0 (0)	0.3
Brainstem functions*	0.1	0.3	
	0 (0)	0 (1)	0.003
Pyramidal functions*	1.1	1.6	
	1 (2)	2 (3)	0.1
Cerebellar functions*	0.8	2.2	
	1 (1)	2 (1)	< 0.001
Sensory functions*	0.4	0.9	
	0 (1)	1 (2)	0.02
Ambulation*	1.1	5.4	
	1 (1)	7 (8)	< 0.001
			
**Lesion volume (mL)**	14.2	77.7	< 0.05

Listed values are the mean group measures where applicable. MS, multiple sclerosis; MS-C, MS control group; MS-T, MS tremor group; SD, standard deviation; RRMS, relapsing remitting multiple sclerosis; PPMS, primary progressive multiple sclerosis; SPMS, secondary progressive multiple sclerosis; DMT, Disease-modifying therapy; EDSS, expanded disability status scale.

^a^ Injectable DMTs: glatiramer acetate, interferon beta-1a, interferon beta-1b, and peginterferon beta-1a.

^b^ Oral DMTs: diroximel fumarate, dimethyl fumarate, teriflunomide, fingolimod and siponimod.

^c^ Infusion DMTs: ocrelizumab, natalizumab, alemtuzumab, rituximab and intravenous immunoglobulin (IVIG).

*Variables were not normally distributed and therefore median (interquartile range) is provided.

In MS-T, 89% of the subjects were right-handed (n = 32), 10 subjects were on tremor medications of which 9 held their medications 24 h prior to assessment, none of the control group subjects were on tremor medications. Data on clinical scores and tremor medications in MS-T are represented in the supplementary, Tables S1 and S2.

### Tract selection

The atlas-based DTI segmentation highlighted 57 WM regions exhibiting significant (*q* < 0.05) group differences in mean diffusivity of which 18 regions correlated with tremor severity, most prominently in the genu of the corpus callosum (left: *r_s_p* = 0.53 and right: *r_s_p* = 0.54), as well as the anterior corona radiata (left: *r_s_p* = 0.53 and right: *r_s_p* = 0.54). TBSS revealed significant widespread group differences in FA, while correlational tractography highlighted specific pathways that correlated with tremor severity, notably the CC-FMN (*t* = 2.10, *q* < 0.001), TR-A-L (*t* = 2.04, *q* < 0.001), CC-FMJ (*t* = 1.94, *q* < 0.001) and the ML-L (*t* = 1.85, *q* < 0.001). The results are summarized in Table 2. The TOIs were constructed by evaluating the significant areas from the different techniques used, which concluded in 53 pathways (Supplementary, Fig S1).

**Table 2.** Tract selection results.

**Atlas-based DTI segmentation^a^**				
ROI	*t*-statistic	*q*-value	*r_s_p*	*q*-value
Anterior CR-L	−2.93	< 0.01	0.53	< 0.05
Anterior CR-R	−3.31	< 0.005	0.54	< 0.05
Anterior Limb IC-R	−4.22	< 0.001	0.42	< 0.05
Body CC-L	−4.52	< 0.001	0.42	< 0.05
Cingulum-L	−3.94	< 0.001	0.43	< 0.05
Cingulum-R	−3.90	0.001	0.47	< 0.05
Genu CC-L	−3.58	0.001	0.53	< 0.05
Genu CC-R	−3.83	0.001	0.54	< 0.05
ICP-L	−4.19	< 0.001	0.43	< 0.05
ICP-R	−3.11	< 0.005	0.44	< 0.05
Posterior CR-L	−4.25	< 0.001	0.48	< 0.05
Posterior CR-R	−3.97	< 0.001	0.51	< 0.05
Posterior TR-L	−3.70	0.001	0.42	< 0.05
Retrolenticular IC-R	−3.96	< 0.001	0.50	< 0.05
Sagittal Stratum-R	−4.08	< 0.001	0.49	< 0.05
SLF-L	−4.80	< 0.001	0.49	< 0.05
SLF-R	−4.53	< 0.001	0.48	< 0.05
Superior CR-R	−4.74	< 0.001	0.45	< 0.05
**TBSS** ^b^				
**MS-C > MS-T at 0.3 FA threshold, *p*-FWE < 0.05**				

CC – Genu CC – Body CC – Splenium CST-R & L ML-R & L ICP-R & L SCP-R & L SLF-R & L	IFOF-R & L UF-R & L IC – Anterior limb R & L IC – Posterior limb R & L IC – Retro lenticular R & L CR – Anterior R & L CR – Superior R & L Fornix R & L	Tapetum R & L Cerebral peduncle R & L CR – Posterior R & L TR – Posterior R & L Sagittal stratum (include ILF and IFOF) R & L External capsule R & L Cingulum (cingulate gyrus/hippocampus) R & L MCP (include Pontine crossing tract)
**Correlational tractography** ^c^		

Tract	mean QA	mean length(mm)	*t*-statistic	*q*-value
CC-FMN	0.29	49.66	2.10	< 0.001
TR-A-L	0.16	48.11	2.04	< 0.001
CC-FMJ	0.40	53.38	1.94	< 0.001
ML-L	0.26	44.34	1.85	< 0.001
TR-S-L	0.17	51.94	1.84	< 0.001
Fornix-L	0.16	52.79	1.80	< 0.001
AF-L	0.24	48.37	1.80	< 0.001
Optic Radiation-L	0.30	46.03	1.70	< 0.001
Fornix-R	0.14	53.29	1.70	< 0.001
SLF2-R	0.26	46.46	1.69	< 0.001
CC-B	0.33	52.67	1.67	< 0.001
CS-P-L	0.29	46.28	1.56	< 0.001
TR-P-L	0.24	51.22	1.56	< 0.001
TR-P-A	0.24	49.65	1.55	< 0.001
CC-T	0.31	57.94	1.45	< 0.001
IFOF-L	0.29	52.55	1.42	< 0.001
ILF-L	0.25	57.52	1.38	< 0.001
IFOF-R	0.29	47.05	1.31	< 0.001
ILF-R	0.24	46.42	1.31	< 0.001

ROI, region on interest; CR, corona radiata (-L: denotes left, -R: denotes right); IC, internal capsule; CC, corpus callosum; TR, thalamic radiation (-A: denotes anterior, -S: denotes superior, and -P: denotes posterior); CST, corticospinal tract; ML, medial lemniscus; ICP, inferior cerebellar peduncle; MCP, middle cerebellar peduncle; SCP, superior cerebellar peduncle; SLF, superior longitudinal fasciculus; IFOF, inferior fronto-occipital fasciculus; ILF, inferior longitudinal fasciculus; UF, uncinate fasciculus; AF, arcuate fasciculus; CC-FMN, forceps minor of corpus callosum; CC-FMJ, forceps major of corpus callosum; CC-B, body of corpus callosum; CC-T, tapetum of corpus callosum.

^a^ Results of atlas-based segmentation for mean diffusivity with *t*-statistic for groupwise comparisons, and *r_s_p* for correlation with TETRAS controlling for age, gender, and SARA.

^b^ TBSS groupwise comparisons results localized using the ICBM-DTI-81 atlas.

^c^ Correlational tractography results correlating with TETRAS controlling for age and gender, *t*-statistic represents strength of correlation.

**Table 3.** Summary of significant correlations between the white matter fiber pathways diffusivity measures and tremor severity.

***FA***				***MD***			
**TETRAS**	*β_LPR_*	*r_s_p*	95% CI	TETRAS	*β_LPR_*	*r_s_p*	95% CI
CC-FMJ	1.38	−0.35*	(−0.64, −0.01)	CS-A-R	7.55	0.4*	(0.03, 0.69)
CC-FMN	−204.98	**−0**.45**	(−0.72, −0.04)	DRTT-R	8.38	0.35*	(−0.11, 0.66)
ML-L	−118.35	**−0**.56***	(−0.75, −0.22)	ML-L	6.33	0.41*	(−0.02, 0.7)
**TETRAS-L**				**TETRAS-L**			
ML-L	−81.4	**−0**.56***	(−0.75, −0.23)	ML-L	2.64	0.4*	(−0.03, 0.66)
**TETRAS-R**				SLF1-L	4.91	0.37*	(−0.02, 0.64)
CC-FMN	−13	−0.35*	(−0.72, 0.1)	**TETRAS-R**			
CS-A-R	−11.18	−0.42*	(−0.71, −0.07)	CS-A-R	4.9	0.35*	(−0.03, 0.68)
**ARCH-L**				**ARCH-L**			
C-L	−1.47	**−0**.53**	(−0.8, −0.18)	AF-R	0.32	0.37*	(0.05, 0.62)
CPT-F-R	−2.89	−0.4*	(−0.65, −0.05)	C-L	0.2	0.36*	(−0.03, 0.66)
CST-L	−2.5	−0.38*	(−0.68, −0.01)	ML-L	0.34	0.35*	(−0.06, 0.65)
SLF1-L	−1.25	−0.36*	(−0.65, 0.02)	**ARCH-R**			
SLF2-R	−0.54	−0.4*	(−0.68, −0.03)	CS-A-L	0.2	0.37*	(0.04, 0.65)
**ARCH-R**				***AD***			
CC-FMN	−1.13	−0.4*	(−0.72, −0.05)	**TETRAS**			
CPT-F-R	−3.21	−0.43*	(−0.69, −0.11)	CS-A-R	7.02	0.4*	(0, 0.7)
CS-S-L	−1.36	**−0**.49**	(−0.75, −0.18)	DRTT-R	7.57	0.39*	(−0.03, 0.68)
DRTT-R	−1.17	−0.34*	(−0.66, 0.04)	ML-L	6.71	0.41*	(−0.01, 0.7)
TR-S-L	−0.26	−0.36*	(−0.66, −0.06)	UF-R	20.54	0.37*	(−0.02, 0.65)
***RD***				**TETRAS-L**			
**TETRAS**				CPT-P-R	0.57	0.35*	(−0.03, 0.62)
CC-FMN	13.8	0.38*	(−0.07, 0.69)	EMC-L	−1.09	0.36*	(−0.02, 0.65)
CS-A-R	7.22	0.39*	(0.01, 0.67)	ML-L	14.04	0.37*	(−0.04, 0.64)
ML-L	6.36	0.44*	(0.02, 0.72)	**TETRAS-R**			
**TETRAS-L**				DRTT-R	5.02	0.4*	(0.02, 0.71)
ML-L	7.53	0.43*	(0.01, 0.66)	**ARCH-L**			
SLF1-L	13.94	0.36*	(−0.02, 0.64)	AF-L	0.49	0.38*	(−0.02, 0.66)
**TETRAS-R**				CST-R	0.16	0.37*	(0.02, 0.64)
CS-A-R	5.15	0.36*	(−0.02, 0.68)	DRTT-L	0.37	0.35*	(−0.03, 0.62)
**ARCH-L**				ML-L	0.37	0.37*	(−0.04, 0.65)
AF-R	0.34	0.38*	(0.07, 0.62)	SLF3-L	0.46	0.36*	(−0.06, 0.64)
C-L	0.25	0.4*	(0.01, 0.7)	**ARCH-R**			
**ARCH-R**				DRTT-R	0.56	0.36*	(−0.05, 0.68)
CS-A-L	0.16	0.38*	(0.09, 0.67)	SLF1-R	−0.07	0.39*	(0.07, 0.73)
CS-A-R	0.37	0.37*	(0.05, 0.68)				
CS-S-L	0.15	0.38*	(0.09, 0.64)				

* *p* < .05, ** *p* < .01, *** *p* < .001, *β_LPR_*; LASSO-partial ridge regression beta coefficient, *r_s_p*; partial regression coefficient, CI, Confidence Interval.

### Tract segmentation

#### Group comparisons

**Figure 3.**
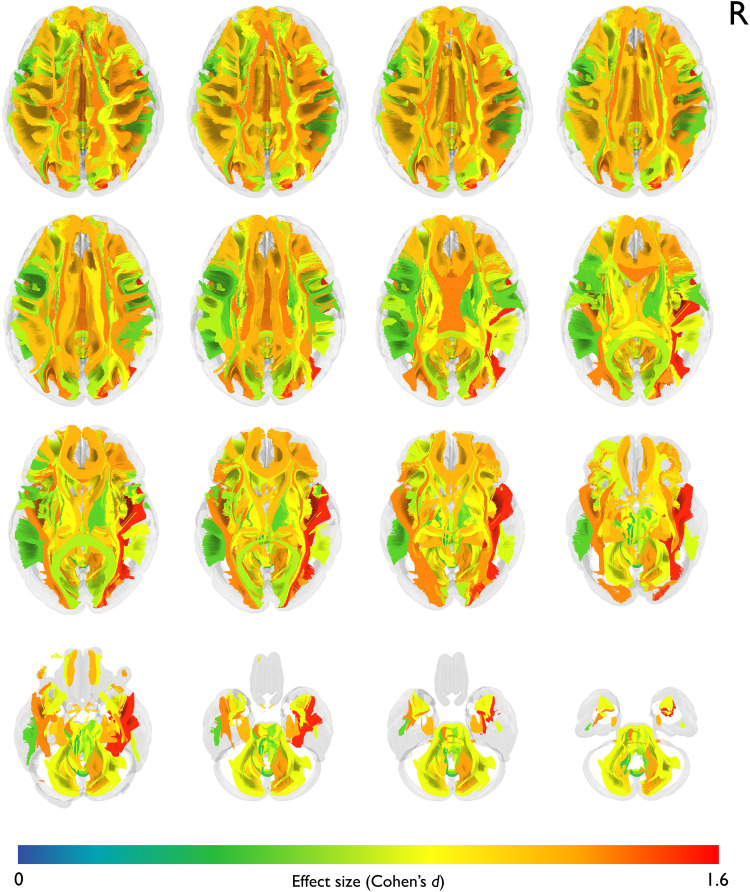
**Group differences in mean tract FA.** Depicting axial slices in tractogram. The pathways are color-coded using their corresponding effect sizes (Cohen's *d*). Effect sizes were calculated via post-hoc comparisons from ANCOVA by comparing tract mean fractional anisotropy between MS-T and MS-C.

A comparison was conducted between the two groups, for the segmented tracts using the mean pathway values of FA, MD, RD, and AD. Group differences in FA are depicted in Figure 3. The greatest reduction in FA in MS-T compared to MS-C was found in the right inferior longitudinal fasciculus (ILF-R) (*d *= 1.53, 95% CI: 0.99–2.07, *q* < 0.001) followed by the left corticospinal tract (CST-L) (*d *= 1.32, 95% CI: 0.80–1.84, *q* < 0.001) and the body of the corpus callosum (CC-B) (*d *= 1.31, 95% CI: 0.79–1.84, *q* < 0.001). Other pathways with significant decreases were the left and right inferior fronto-occipital fasciculus (IFOF-L, IFOF-R), and the left inferior longitudinal fasciculus (ILF-L).

**Figure 4.**
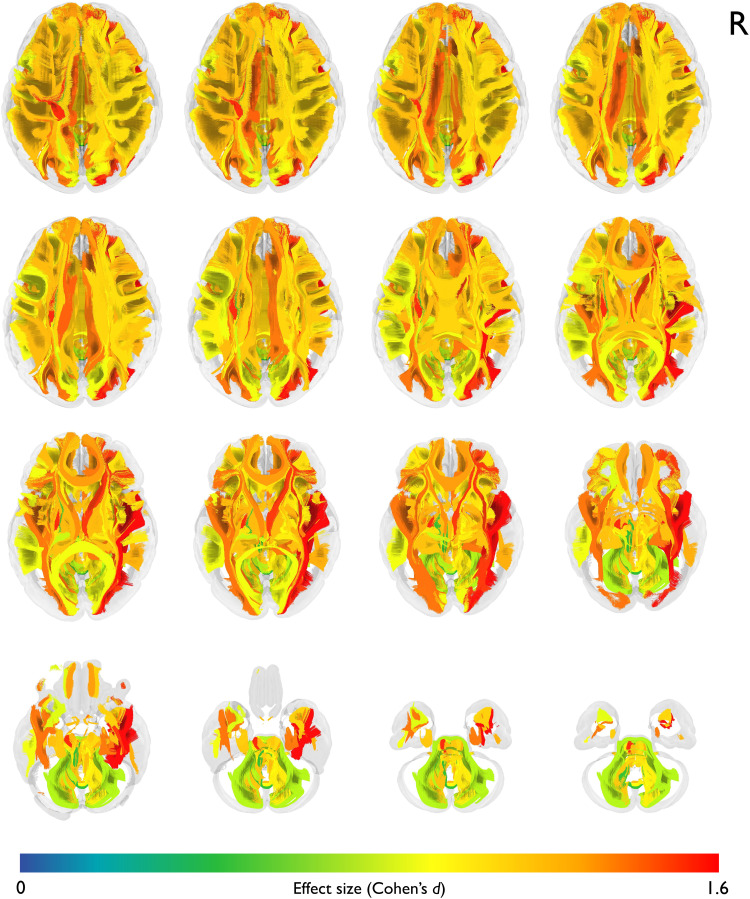
**Group differences in mean tract MD.** Depicting axial slices in tractogram. The pathways are color-coded using their corresponding effect sizes (Cohen's *d*). Effect sizes were calculated via post-hoc comparisons from ANCOVA by comparing tract mean diffusivity between MS-T and MS-C.

**Figure 5.**
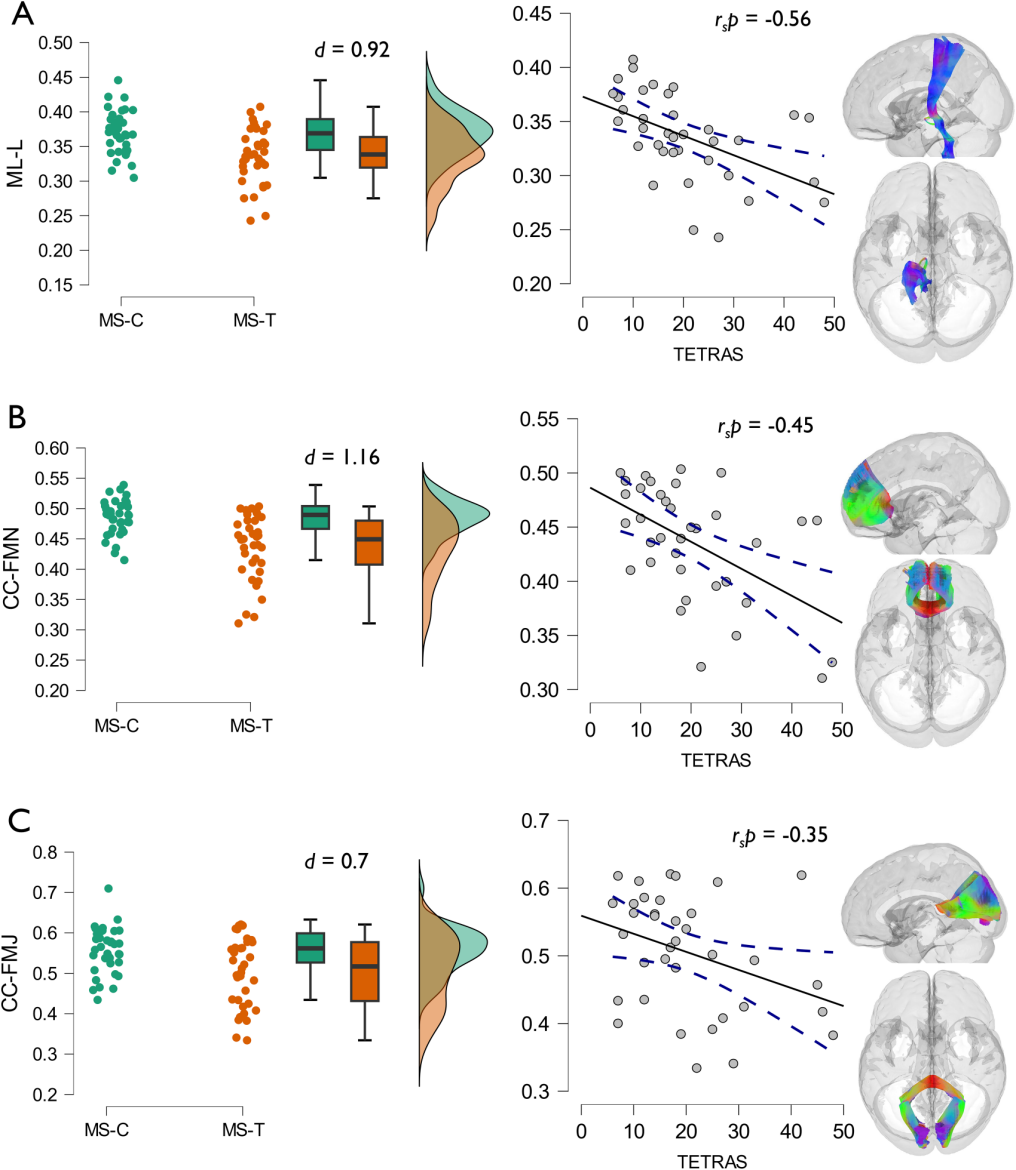
**Correlations between mean tract FA and tremor severity.** Raincloud plots represent the distribution of measures for both groups (MS-C in green and MS-T in orange). Effect size (*d*) calculated via post-hoc comparisons from ANCOVA comparing mean tract fractional anisotropy. Scatter plots showing the relationship between tremor severity and mean tract FA. Blue dashed lines represent 95% confidence interval. Partial correlation coefficients (*r_s_p*) calculated using Spearman's rank correlation. Tremor severity measured by TETRAS. Panel A: left medial lemniscus (ML-L). Panel B: forceps minor of corpus callosum (CC-FMN). Panel C: forceps major of corpus callosum (CC-FMJ).

In terms of MD, the most prominent increase in MS-T compared to MS-C was again seen in the ILF-R (*d *= -1.6, 95% CI: −2.15 – −1.06, *q* < 0.001), followed by the CST-L (*d *= -1.52, 95% CI: −2.06 – −0.98, *q* < 0.001) and the IFOF-R (*d* = -1.49, 95% CI: −2.03 - −0.96, *q* < 0.001). Other pathways with significant increases included the right anterior thalamic radiation (TR-A-R), followed by the parietal section of the left superior longitudinal fasciculus, and the IFOF-L as depicted in Figure 4. Group differences in FA and MD for all 53 pathways are listed in the supplementary, Table S3.

A similar pattern was observed for RD, with the ILF-R (*d *= -1.65, 95% CI: −2.2 – −1.1, *q* < 0.001), followed by the CST-L (*d *= -1.54, 95% CI: −2.08 – −1, *q* < 0.001), and the IFOF-R (*d* = -1.53, 95% CI: −2.07 - −0.99, *q* < 0.001). The greatest increases in AD were observed in the TR-A-R (*d *= -1. 5, 95% CI: −2.03 – −0.96, *q* < 0.001), followed by the CST-L (*d *= -1.43, 95% CI: −1.96 – −0.9, *q* < 0.001) and the ILF-R (*d *= -1.34, 95% CI: −1.87 – −0.82, *q* < 0.001). Interestingly, the left dentatorubrothalamic tract was the only pathway that did not exhibit significant group differences for any of the diffusivity measures (FA: *d* = 0.37, *q* = 0.11, MD: *d* = -0.4, *q* = 0.09, RD: *d* = -0.39, *q* = 0.87, and AD: *d* = -0.41, *q* = 0.09).

#### Correlations with tremor severity

All 53 segmented tracts were evaluated. The correlations between tremor severity measurements and the diffusivity measures of the pathways were calculated while controlling for the effect of ataxia, age, and gender. A significant negative correlation between FA and TETRAS was noted in the left medial lemniscus (ML-L) (*r_s_p* = -0.56, 95% CI: −0.75, −0.22, *p *< 0.001), and the forceps minor of corpus callosum (CC-FMN) (*r_s_p* = -0.45, 95% CI: −0.72, −0.04, *p *< 0.01), followed by the forceps major of corpus callosum (CC-FMJ) (*r_s_p* = -0.35, 95% CI: −0.64, −0.01, *p *= 0.05) as shown in Figure 5.

The left component of TETRAS correlated negatively with the FA of the ML-L (*r_s_p* = -0.56, 95% CI: −0.75, −0.23, *p *< 0.001). Meanwhile, the right component of TETRAS correlated negatively with the FA of the right anterior cortico-striatal tract (CS-A-R) (*r_s_p* = -0.42, 95% CI: −0.7, −0.07, *p *< 0.05) and the CC-FMN (*r_s_p* = -0.35, 95% CI: −0.72, −0.1, *p *= 0.05). The scores of the left Archimedean spiral correlated negatively with the FA of the left cingulum (*r_s_p* = -0.53, 95% CI: −0.8, −0.18, *p *= 0.001) and the right frontal cortico-pontine tract (CPT-F-R) (*r_s_p* = -0.4, 95% CI: −0.65, −0.05, *p *< 0.05), while the scores of the right Archimedean spiral correlated negatively with the left superior cortico-striatal tract (CS-S-L) (*r_s_p* = -0.49, 95% CI: −0.75, −0.18, *p *< 0.01), the CPT-F-R (*r_s_p* = -0.43, 95% CI: −0.69, −0.1, *p *= 0.01) and the CC-FMN (*r_s_p* = -0.4, 95% CI: −0.72, −0.05, *p *< 0.05).

To explore the influence of laterality and handedness we interchanged the left and right medial lemniscus FA measures for left-handed participants (n = 4). Interestingly, this modification revealed a slightly stronger correlation with TETRAS (*r_s_p* = -0.59, *p* < 0.001).

Looking at the diffusivities, positive correlations with TETRAS were found in the ML-L (MD: *r_s_p* = 0.41, 95% CI: −0.02, 0.7, *p *< 0.05, RD: *r_s_p* = 0.44, 95% CI: 0.02, 0.72, *P *= 0.01 and AD: *r_s_p* = 0.41, 95% CI: −0.01, 0.7, *p *< 0.05), the right anterior corticostriatal tract (MD: *r_s_p* = 0.4, 95% CI: 0.03, 0.7, *p *< 0.05, RD: *r_s_p* = 0.39, 95% CI: 0.01, 0.67, *p *< 0.05 and AD: *r_s_p* = 0.4, 95% CI: 0, 0.7, *p *< 0.05), as well as the forceps minor (RD: *r_s_p* = 0.38, 95% CI: −0.07, 0.69, *p *< 0.05). Significant correlations are listed in Table 3.

## Discussion

Our results showed significant differences in diffusion metrics within several pathways in MS-T compared to MS-C. The greatest reduction in FA was seen in the right inferior longitudinal fasciculus, followed by the left corticospinal tract, and body of corpus callosum. In terms of MD and RD, the greatest increase was again seen in the right inferior longitudinal fasciculus, followed by the left corticospinal tract and right inferior fronto-occipital fasciculus, suggesting that demyelination largely drives dysfunction within several brain pathways. Interestingly, correlations between tremor severity and diffusivity measures for FA, MD, RD, and AD were seen in the left medial lemniscus, the forceps major of corpus callosum, and the forceps minor of corpus callosum, which all showed significant group differences, as well. Indeed, correlations between TETRAS and FA were stronger for the medial lemniscus when transposing the right-left brain orientation of left-handed participants. We hypothesize, in light of our findings, that MS-related tremor associates with widespread structural disconnectivity secondary to lesion-driven inflammation, especially in regions traversed by projection pathways and consequent axonal and possibly transsynaptic^32^ degeneration, while it's severity may be modulated by dysfunction of specific WM pathways.

The medial lemniscus is part of the dorsal column-medial lemniscus system, which is responsible for relaying the peripheral mechanoreceptive sensations.^33^ Heenan et al.^34^ suggested that, mechanistically, MS-related tremor is secondary to sensory feedback maladaptation and defects in internal estimates of limb dynamics. Indeed, peripheral limb cooling was shown in one study to reduce tremor in PwMS, though the mechanisms remain unexplored.^35^ Feys et al.^36^ reported correlations between tremor amplitude and lesion load in the contralateral pons, suggesting the possible involvement of the descending rubro-olivary pathway. Lesions in the midbrain can cause dystonia frequently associated with tremor, while thalamic or striatopallidal lesions can cause pure focal dystonia. A study showed that the resting and postural components of dystonia-related tremor occurred exclusively with lesions disrupting the dentato-rubro-olivary loop as well as the medial lemniscus.^37^

Therefore, two potential explanations exist for the strong correlation between tremor severity and the medial lemniscus diffusivity measures in our sample. One explanation could be the involvement of the dentato-rubro-olivary circuit^38^ in MS-related tremor due to its intimate anatomical proximity.^33^ The second explanation could be a reduction in sensory afferents modulating movement,^39,40^ as the medial lemniscus is the main pathway for proprioception and conveys sensory information to various circuits to regulate smooth motor action by providing positional and gravitational information.

### Limitations

Our study has some limitations, most notably the absence of a validated MS-related tremor scale to precisely characterize and measure tremor severity. TETRAS offers improved characterization of severe tremor and correlates well with other scoring systems previously usurped to quantify MS-related tremor, such as the Fahn-Tolosa-Marin scale.^15,16^ EDSS differences between groups were primarily driven by higher cerebellar and ambulation subscores, due to the need for assistance devices secondary to ataxia, though all correlations were adjusted for SARA. Diffusion MRI fiber tracking is a user-defined process, which might affect reproducibility of results. We mitigated this issue methodologically by using a semi-automated approach to standardize the procedure.^26,28^ Another limitation is that our DTI sequence is not optimized fully for GQI-based reconstruction, although it has been validated on a similar acquisition protocol.^30^ Additionally, in this initial work, we did not explore in-depth the impact of MS-related lesions, either in the brain or spinal cord, on the described tracts. Lastly, inferences from our findings are restricted by the limited sample size and the exploratory nature of the study.

## Conclusions

The results showed significant widespread changes in diffusivity measures along several association, projection, and commissural pathways associated with MS-related tremor. The greatest dysfunction between groups was seen in the inferior longitudinal fasciculus followed by the corticospinal tract. In the tremor group, we found a correlation between tremor severity and diffusivity measures of certain projection and commissural pathways. The correlations between tremor severity and diffusivity measures in the medial lemniscus might suggest a potential involvement of the dentato-rubro-olivary circuit in MS-related tremor or a reduction in sensory afferents affecting movement due to the medial lemniscus being the main pathway for proprioception. Further confirmatory studies with larger sample sizes are needed to validate the findings, studying both causality and temporality between tract-specific dysfunction and tremor incidence as well as severity.

## Supplemental Material

sj-pdf-1-mso-10.1177_20552173231208271 - Supplemental material for Identifying the white matter pathways involved in multiple sclerosis-related tremor using diffusion tensor imaging Click here for additional data file. Supplemental material, sj-pdf-1-mso-10.1177_20552173231208271 for Identifying the white matter pathways involved in multiple sclerosis-related tremor using diffusion tensor imaging by Ahmed Bayoumi, Khader M. Hasan, Jorge Patino, Zafer Keser, Joseph A. Thomas, Refaat E. Gabr, Claudia Pedroza, Arash Kamali, Mya C. Schiess, Jerry S. Wolinsky and John A. Lincoln in Multiple Sclerosis Journal – Experimental, Translational and Clinical

sj-pdf-2-mso-10.1177_20552173231208271 - Supplemental material for Identifying the white matter pathways involved in multiple sclerosis-related tremor using diffusion tensor imaging Click here for additional data file. Supplemental material, sj-pdf-2-mso-10.1177_20552173231208271 for Identifying the white matter pathways involved in multiple sclerosis-related tremor using diffusion tensor imaging by Ahmed Bayoumi, Khader M. Hasan, Jorge Patino, Zafer Keser, Joseph A. Thomas, Refaat E. Gabr, Claudia Pedroza, Arash Kamali, Mya C. Schiess, Jerry S. Wolinsky and John A. Lincoln in Multiple Sclerosis Journal – Experimental, Translational and Clinical
