# Supplementary material for: Identifying the white matter pathways involved in multiple sclerosis-related tremor using diffusion tensor imaging
Source: Mult Scler J Exp Transl Clin. 2023 Nov 8;9(4):20552173231208271. doi: 10.1177/20552173231208271 (PMC10631316; doi:10.1177/20552173231208271)
Supplement: sj-pdf-1-mso-10.1177_20552173231208271 - Supplemental material for Identifying the white matter pathways involved in multiple sclerosis-related tremor using diffusion tensor imaging [file sj-pdf-1-mso-10.1177_20552173231208271.pdf]

Identifying the white matter pathways involved in multiple sclerosis-related tremor using diffusion tensor imaging

Supplementary material

Contents

I. Tables 2

II. Figure 6

III. Methods 7

Image acquisition 7

DTI data processing 7

Image analysis 8

A. Tract selection 8

B. Tract segmentation 10

IV. References 11

I. Tables

Table S1. Tremor severity measures in the MS-related tremor group MS-T.

Subject	Age	Gender	Handedness	TETRAS	TETRAS-L	TETRAS-R	SARA	ARCH-L	ARCH-R
**1**	24.2	male	left	11	0	11	17.5	1	2.5
**2**	49.6	male	right	46	11	23	21.5	3	4
**3**	49	male	right	27	12	10	24	3.5	3
**4**	59.3	female	right	10	6	3	2	2	1
**5**	44.4	female	right	20	3	10	20.5	2	2.5
**6**	49	female	right	12	3	8	3	2	2
**7**	62.2	female	right	26	12	12	7	2	2
**8**	48.8	female	right	21	10	9	3	2.5	2
**9**	28.9	male	right	17	5	11	19	2	2.5
**10**	39	female	right	16	7	5	10.5	2	1.5
**11**	45.5	male	right	7	3	3	12	2	2
**12**	48.2	male	right	14	6	6	10.5	1.5	1.5
**13**	56.8	female	right	18	10	5	10.5	2	2
**14**	54	female	right	14	3	7	18.5	2	2.5
**15**	43.2	male	right	17	6	5	16	2	1.5
**16**	37.9	male	right	7	2	5	1	1.5	1.5
**17**	43.5	female	left	31	8	20	9	2	3
**18**	48.6	female	right	12	3	8	2.5	2	2
**19**	63.2	male	right	20	10	8	8	1.5	1.5
**20**	66.5	male	right	10	3	6	4	2	1.5
**21**	67.3	male	right	25	12	7	17.5	3.5	2.5
**22**	42.1	female	right	14	7	6	15.5	2	1.5
**23**	57.7	female	right	42	18	15	26	3.5	2
**24**	50.3	female	right	33	14	11	22	3	2
**25**	52.3	female	right	7	7	0	3	2.5	2
**26**	50.2	male	right	18	5	10	6	2	3
**27**	39	male	left	18	6	11	17	1.5	2
**28**	40.3	female	right	18	8	8	15.5	1.5	1.5
**29**	40.2	female	left	29	11	13	15.5	2	3
**30**	24.7	female	right	22	14	6	27.5	3.5	1
**31**	34.3	male	right	48	21	20	10	2.5	3.5
**32**	28.3	female	right	46	19	18	25	4	3.5
**33**	39	male	right	6	5	1	9.5	2	1.5
**34**	44.4	female	right	8	1	6	12	2	2
**35**	44.3	female	right	25	12	9	21	3	1.5
**36**	65.6	female	right	12	5	5	3.5	2	1.5

MS = multiple sclerosis; TETRAS = The Essential Tremor Rating Assessment Scale; SARA = Scale for Assessment and Rating of Ataxia; ARCH = Archimedean spiral scores.

Table S2. Tremor medications in MS-T.

Subject	Medication	Last Dose $\geq$ 24 hours from visit date
**1**	Propranolol	yes
**2**	-	-
**3**	Primidone	yes
**4**	-	-
**5**	Cannabidiol oil	yes
**6**	-	-
**7**	-	-
**8**	Clonazepam	yes
**9**	-	-
**10**	-	-
**11**	-	-
**12**	-	-
**13**	-	-
**14**	Cyclobenzaprine	yes
**15**	-	-
**16**	-	-
**17**	-	-
**18**	Propranolol	yes
**19**	-	-
**20**	-	-
**21**	-	-
**22**	-	-
**23**	Primidone	yes
**24**	-	-
**25**	Metoprolol	yes
**26**	-	-
**27**	-	-
**28**	-	-
**29**	Valproic acid	No, taken morning of visit date
**30**	-	-
**31**	-	-
**32**	-	-
**33**	-	-
**34**	Baclofen	yes
**35**	-	-
**36**	-	-

Table S3. Differences in white matter tracts mean FA and MD between MS-C and MS-T, effect sizes are expressed in terms of Cohen's *d*, *p*-values were corrected for false discovery rate (*q*-value).

**Tract**	**FA**		**MD**	
	**Cohen's *d* (95% CI)**	***q*-value**	**Cohen's *d* (95% CI)**	***q*-value**
**AF-L**	0.53 (0.05, 1.01)	0.028798	-0.82 (-1.31, -0.33)	0.000985
**AF-R**	0.76 (0.27, 1.24)	0.002791	-1.19 (-1.7, -0.68)	0.000013
**C-L**	1.16 (0.65, 1.67)	0.000029	-1.08 (-1.59, -0.57)	0.000040
**C-R**	1.19 (0.68, 1.71)	0.000020	-1.33 (-1.86, -0.81)	0.000002
**CC-B**	1.31 (0.79, 1.84)	0.000008	-1.02 (-1.52, -0.51)	0.000089
**CC-FMJ**	0.7 (0.21, 1.19)	0.005224	-0.82 (-1.31, -0.33)	0.000946
**CC-FMN**	1.16 (0.65, 1.67)	0.000030	-1.24 (-1.76, -0.72)	0.000007
**CC-T**	0.84 (0.35, 1.34)	0.001132	-1.1 (-1.61, -0.6)	0.000032
**CPT-F-L**	0.86 (0.37, 1.35)	0.000969	-1.15 (-1.67, -0.64)	0.000020
**CPT-F-R**	0.62 (0.14, 1.1)	0.012341	-1.08 (-1.59, -0.58)	0.000041
**CPT-O-L**	0.6 (0.12, 1.08)	0.015458	-0.89 (-1.38, -0.39)	0.000405
**CPT-O-R**	0.83 (0.34, 1.32)	0.001335	-1.04 (-1.54, -0.54)	0.000067
**CPT-P-L**	0.49 (0.01, 0.96)	0.043783	-0.97 (-1.47, -0.47)	0.000148
**CPT-P-R**	0.67 (0.18, 1.15)	0.007256	-1.2 (-1.71, -0.69)	0.000012
**CST-L**	1.32 (0.8, 1.84)	0.000011	-1.52 (-2.06, -0.98)	0.000000
**CST-R**	0.8 (0.31, 1.29)	0.001765	-1 (-1.51, -0.5)	0.000098
**CS-A-L**	0.76 (0.27, 1.25)	0.002787	-0.95 (-1.44, -0.45)	0.000194
**CS-A-R**	0.97 (0.47, 1.47)	0.000254	-0.99 (-1.49, -0.49)	0.000114
**CS-P-L**	1.13 (0.62, 1.64)	0.000041	-1.19 (-1.7, -0.67)	0.000013
**CS-P-R**	0.89 (0.4, 1.39)	0.000673	-1.19 (-1.71, -0.68)	0.000013
**CS-S-L**	0.67 (0.19, 1.16)	0.007117	-1.13 (-1.64, -0.62)	0.000022
**CS-S-R**	0.82 (0.32, 1.31)	0.001569	-0.95 (-1.44, -0.45)	0.000190
**DRTT-L**	0.37 (-0.1, 0.85)	0.118450	-0.4 (-0.88, 0.07)	0.090919
**DRTT-R**	0.59 (0.11, 1.07)	0.016585	-0.88 (-1.37, -0.38)	0.000470
**EMC-L**	0.97 (0.47, 1.47)	0.000244	-1.34 (-1.86, -0.81)	0.000002
**EMC-R**	1.12 (0.61, 1.63)	0.000043	-1.31 (-1.83, -0.79)	0.000003
**FX**	1.03 (0.53, 1.53)	0.000108	-1.11 (-1.62, -0.6)	0.000030
**ICP-L**	1.06 (0.55, 1.56)	0.000079	-1.1 (-1.61, -0.59)	0.000032
**ICP-R**	1.24 (0.72, 1.75)	0.000011	-0.95 (-1.45, -0.45)	0.000185
**IFOF-L**	1.3 (0.78, 1.82)	0.000008	-1.35 (-1.87, -0.83)	0.000002
**IFOF-R**	1.25 (0.73, 1.77)	0.000012	-1.49 (-2.03, -0.96)	0.000000
**ILF-L**	1.29 (0.77, 1.81)	0.000007	-1.34 (-1.86, -0.81)	0.000002
**ILF-R**	1.53 (0.99, 2.07)	0.000001	-1.6 (-2.15, -1.06)	0.000000
**ML-L**	0.92 (0.43, 1.42)	0.000457	-0.98 (-1.48, -0.48)	0.000127
**ML-R**	0.81 (0.32, 1.3)	0.001567	-1.01 (-1.51, -0.51)	0.000096
**MCP**	0.79 (0.3, 1.28)	0.001985	-0.7 (-1.18, -0.21)	0.004351
**RT-L**	0.89 (0.4, 1.38)	0.000668	-1.05 (-1.56, -0.55)	0.000055

**RT-R**	0.67 (0.18, 1.15)	0.007345	-0.98 (-1.48, -0.48)	0.000123
**SCP**	0.75 (0.26, 1.24)	0.002927	-1.14 (-1.65, -0.63)	0.000024
**SLF1-L**	1.08 (0.57, 1.59)	0.000063	-1.39 (-1.92, -0.86)	0.000001
**SLF1-R**	0.94 (0.44, 1.44)	0.000358	-1.01 (-1.51, -0.51)	0.000091
**SLF2-L**	1.1 (0.59, 1.61)	0.000050	-1.13 (-1.64, -0.62)	0.000023
**SLF2-R**	1.24 (0.72, 1.75)	0.000013	-0.99 (-1.49, -0.49)	0.000111
**SLF3-L**	0.71 (0.22, 1.2)	0.004733	-0.93 (-1.43, -0.43)	0.000222
**SLF3-R**	0.55 (0.07, 1.03)	0.023418	-1.08 (-1.59, -0.57)	0.000039
**TR-A-L**	1.05 (0.55, 1.55)	0.000086	-1.28 (-1.79, -0.76)	0.000004
**TR-A-R**	1.11 (0.6, 1.61)	0.000051	-1.49 (-2.03, -0.96)	0.000000
**TR-P-L**	1.07 (0.56, 1.57)	0.000073	-0.74 (-1.23, -0.25)	0.002657
**TR-P-R**	1.11 (0.6, 1.61)	0.000048	-0.94 (-1.44, -0.44)	0.000201
**TR-S-L**	0.89 (0.39, 1.38)	0.000659	-1.13 (-1.64, -0.63)	0.000024
**TR-S-R**	0.53 (0.05, 1.01)	0.028718	-1.13 (-1.64, -0.62)	0.000024
**UF-L**	0.78 (0.29, 1.27)	0.002150	-0.77 (-1.26, -0.28)	0.001872
**UF-R**	0.8 (0.31, 1.3)	0.001696	-1.04 (-1.55, -0.54)	0.000064

MS-C = MS control group; MS-T = MS tremor group; FA = fractional anisotropy; MD = mean diffusivity; CI = confidence interval; AF = arcuate fasciculus (L suffix denotes left and R suffix denotes right, e.g., AF-L and AF-R respectively); C = cingulum; CC-B = body of the corpus callosum; CC-FMJ = forceps minor of the corpus callosum; CC-FMN = forceps minor of the corpus callosum; CC-T = tapetum of the corpus callosum; CPT-F = frontal corticopontine tract; CPT-O = occipital corticopontine tract; CPT-P = parietal corticopontine tract; CS-A = anterior corticostriatal tract; CS-P = posterior corticostriatal tract; CS-S = superior corticostriatal tract; CST = corticospinal tract; DRTT = dentatorubrothalamic tract; EMC = extreme capsule; FX = fornix; ICP = inferior cerebellar peduncle; IFOF = inferior fronto-occipital fasciculus; ILF = inferior longitudinal fasciculus; MCP = middle cerebellar peduncle; ML = medial lemniscus; RT = reticular tract; SARA = Scale for Assessment and Rating of Ataxia; SCP = superior cerebellar peduncle; SLF1 = superior longitudinal fasciculus 1; SLF2 = dorsal superior longitudinal fasciculus; SLF3 = ventral superior longitudinal fasciculus; TR-A = anterior thalamic radiation; TR-P = posterior thalamic radiation; TR-S = superior thalamic radiation; UF = uncinate fasciculus.

II. Figure

Figure S1. Tracts-of-interest (TOIs) selected by our preliminary analysis (tract selection).

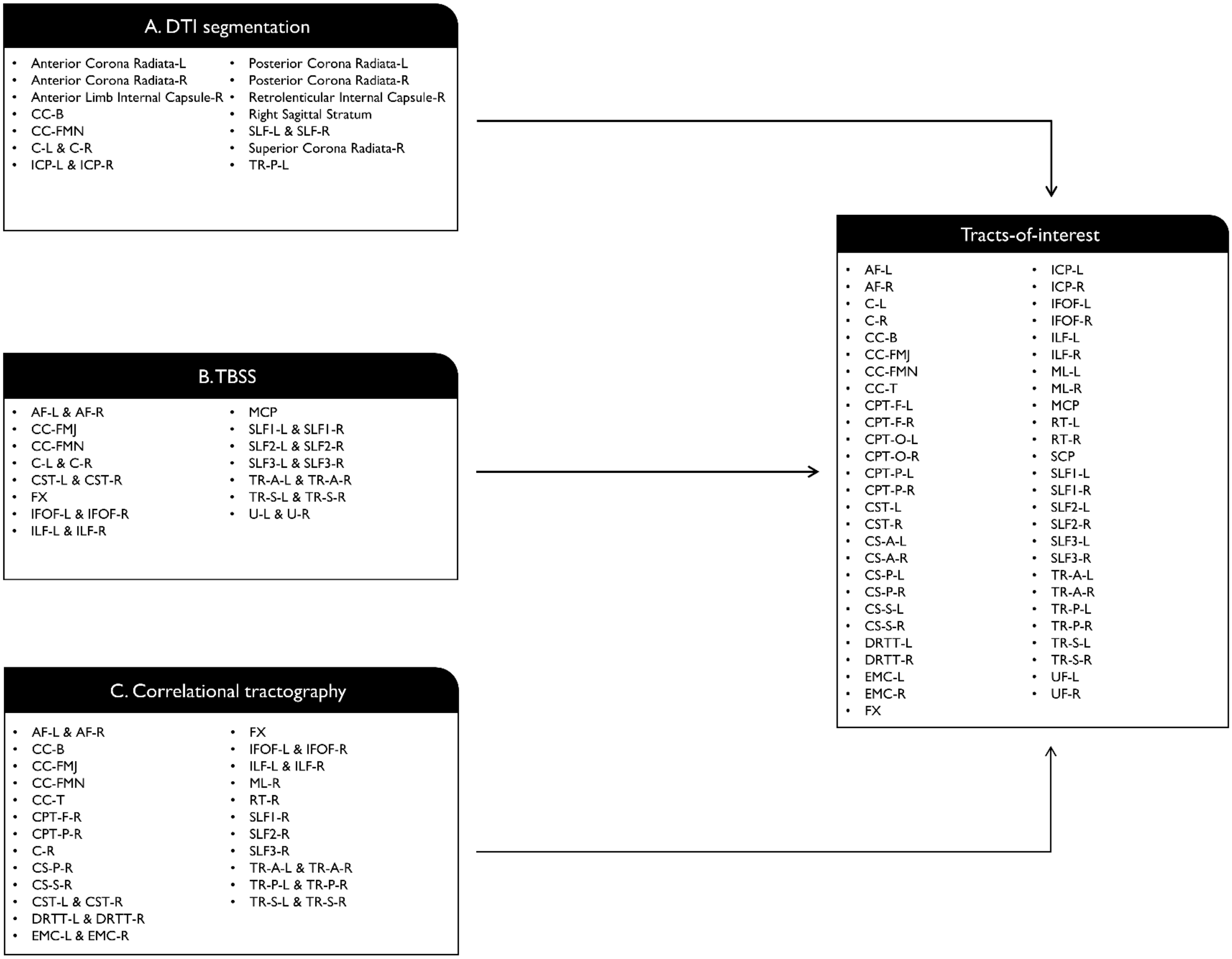

DTI = Diffusion tensor imaging; TBSS = tract-based spatial statistics.

III. Methods

Image acquisition

MRI scans were performed using a 3.0 T Philips Ingenia research scanner with a maximum gradient amplitude of 45 mT/m and a 15-channel SENSE-compatible head coil (Philips Medical Systems, Best, Netherlands). High-resolution 3D T1-weighted magnetization prepared rapid gradient echo (MPRAGE) images (voxel size: $1 \times 1 \times 1$ mm^3, field of view (FOV): 256×256 mm^2, repetition time (TR)/ time to echo (TE): 8/3.7 ms), used for anatomical registration, were acquired at the start of each scanning session, as well as T2-weighted fluid-attenuated inversion recovery (FLAIR) images (voxel size: $1 \times 1 \times 1$ mm^3, FOV: 256×256 mm^2, TR/TE: 4800/300 ms), which were used for lesion segmentation. Additionally, diffusion-tensor images (DTI) were obtained using a single-shot spin-echo diffusion-sensitized echo-planar imaging sequence with a balanced Icosa21 tensor encoding scheme.[1] The b-factor was 1,000 s mm^{-2}, TR/TE 7,100/65 ms, FOV = 256×256 mm, and the slice thickness was 3 mm with a 0 mm gap and a total of 44 slices.

DTI data processing

The DWI data were intra-registered to the baseline “b0” images (without diffusion weighting) to correct for the eddy-current image distortions in each of the 21 diffusion-weighted volumes using a mutual information approach.[2] Further post-processing is described in these earlier works.[3, 4]

Image analysis

A. Tract selection

1. Atlas-based DTI Segmentation: *DTI Studio* was used to decode and extract tensor data from DTI sequences. MRICloud was used to perform DTI-based segmentation for 168 brain regions that include both grey and white matter regions.

- Type: Whole brain
- Output: Mean Fractional Anisotropy (FA), mean diffusivity (MD), axial diffusivity (AD) and radial diffusivity (RD)
- Software: *DTI Studio* (version 3.0.2), MRICloud[5]

2. Tract-based spatial statistics: in FMRIB Software Library, TBSS[6] was used to compare the mean FA skeleton between MS-T and MS-C. TBSS uses nonlinear registration followed by projection onto an alignment-invariant tract representation creating a mean FA skeleton. The individual FA images were first registered and transformed into a standard space. A mean FA image and skeleton were then created for each group by combining the transformed images. We conducted *voxelwise* statistics to compare the group skeletons and identify any significant differences. Using *atlasquery*, the identified voxels were finally localized on the ICBM-DTI-81 white-matter labels atlas which includes 48 white matter tract labels created by manual segmentation of a standard-space average of diffusion MRI tensor maps from 81 healthy subjects.

- Type: White matter
- Output: group FA maps and skeletons representing mean FA values to estimate statistical group differences in white matter FA
- Software: *FMRIB Software Library (FSL)* (version 6.0)

3. Correlational Tractography: In MS-T, the diffusion data were reconstructed in the Montreal Neurological Institute template space using q-space diffeomorphic reconstruction[7] to obtain the spin distribution function.[8] For quality assurance, consistency in image dimension, resolution, DWI count, and shell count was checked. Neighboring DWI correlation, which decreases with defects in diffusion signals, was used to ensure the lack of low-quality outliers.[9] This analysis was conducted using quantitative anisotropy (QA)[10] and FA separately as a tracking index and TETRAS as the study variable while controlling for the effects of age and gender. A nonparametric Spearman correlation was used to derive the correlation with a T-score threshold of 2.5 and a length threshold of 20 voxels for the selected tracts. The tracks were filtered by topology-informed pruning[11] with 4 iterations. To estimate the false discovery rate, a total of 10,000 randomized permutations were performed to obtain the null distribution of the track length.

- Type: White matter
- Output: Tract segments with anisotropy correlating with tremor severity
- Software: *DSI Studio (version 12/22/2022)*

This technique infers the statistical association with the study variable within white matter pathways by analyzing the local connectomes. The local connectome refers to the connectivity between adjacent voxels in a white matter fascicle as determined by the density of the diffusing spins. This is achieved by reconstructing the diffusion MRI data into a common stereotaxic space.[7, 8] By using permutation tests on the length of coherent associations, significant connections can be identified and tracked along the white matter pathways. Connectometry is a more sensitive method compared to region-of-interest approaches.[12]

B. Tract segmentation

The diffusion data were reconstructed using generalized q-sampling imaging[8] with a diffusion sampling length ratio of 2. The diffusion sampling length ratio was set by visual inspection of the fiber directions at the mid and lateral corpus callosum ensuring a homogenous direction in the mid corpus callosum and resolution of crossing patterns laterally. The accuracy of b-table orientation was examined by comparing fiber orientations with those of a population-averaged template.[13] The pre-constructed seed and target regions in atlas space[14] were transformed into the subject's DWI space for tractography. The output was inspected for an accurate representation of the neuroanatomical topology.[14] The tracking tolerance was set to a Hausdorff distance of 16 mm and the findings underwent 16 iterations of topology-informed pruning to remove possible false connections.[11] The tracking index used was the normalized QA value, with a random tracking threshold between 0.5 and 0.7 of Otsu's threshold, an angular threshold was randomly chosen between 15 and 90 degrees, and a step-size was randomly chosen between 0.5 and 1.5 voxel distances. The minimum and maximum track length were set to 20 mm and 300 mm, respectively.

- Type: White matter tractography
- Output: mean tract diffusivity measures (FA, MD, AD, and RD)
- Software: *DSI Studio (version 12/22/2022)*

IV. References

1. Hasan KM and Narayana PA. Computation of the fractional anisotropy and mean diffusivity maps without tensor decoding and diagonalization: Theoretical analysis and validation. *Magn Reson Med* 2003; 50: 589-598. DOI: 10.1002/mrm.10552.
2. Netsch T and van Muiswinkel A. Quantitative evaluation of image-based distortion correction in diffusion tensor imaging. *IEEE Trans Med Imaging* 2004; 23: 789-798. DOI: 10.1109/tmi.2004.827479.
3. Hasan KM. A framework for quality control and parameter optimization in diffusion tensor imaging: theoretical analysis and validation. *Magn Reson Imaging* 2007; 25: 1196-1202. 20070418. DOI: 10.1016/j.mri.2007.02.011.
4. Hasan KM, Kamali A, Abid H, et al. Quantification of the spatiotemporal microstructural organization of the human brain association, projection and commissural pathways across the lifespan using diffusion tensor tractography. *Brain Structure and Function* 2010; 214: 361-373. DOI: 10.1007/s00429-009-0238-0.
5. Mori S, Wu D, Ceritoglu C, et al. MRICloud: Delivering High-Throughput MRI Neuroinformatics as Cloud-Based Software as a Service. *Computing in Science & Engineering* 2016; 18: 21-35. DOI: 10.1109/MCSE.2016.93.
6. Smith SM, Jenkinson M, Johansen-Berg H, et al. Tract-based spatial statistics: Voxelwise analysis of multi-subject diffusion data. *NeuroImage* 2006; 31: 1487-1505. DOI: https://doi.org/10.1016/j.neuroimage.2006.02.024.
7. Yeh FC and Tseng WY. NTU-90: a high angular resolution brain atlas constructed by q-space diffeomorphic reconstruction. *Neuroimage* 2011; 58: 91-99. 20110616. DOI: 10.1016/j.neuroimage.2011.06.021.
8. Yeh FC, Wedeen VJ and Tseng WY. Generalized q-sampling imaging. *IEEE Trans Med Imaging* 2010; 29: 1626-1635. 20100318. DOI: 10.1109/tmi.2010.2045126.
9. Yeh F-C, Zaydan IM, Suski VR, et al. Differential tractography as a track-based biomarker for neuronal injury. *NeuroImage* 2019; 202: 116131. DOI: https://doi.org/10.1016/j.neuroimage.2019.116131.
10. Yeh F-C, Verstynen TD, Wang Y, et al. Deterministic Diffusion Fiber Tracking Improved by Quantitative Anisotropy. *PLOS ONE* 2013; 8: e80713. DOI: 10.1371/journal.pone.0080713.
11. Yeh FC, Panesar S, Barrios J, et al. Automatic Removal of False Connections in Diffusion MRI Tractography Using Topology-Informed Pruning (TIP). *Neurotherapeutics* 2019; 16: 52-58. DOI: 10.1007/s13311-018-0663-y.
12. Yeh FC, Badre D and Verstynen T. Connectometry: A statistical approach harnessing the analytical potential of the local connectome. *Neuroimage* 2016; 125: 162-171. 20151021. DOI: 10.1016/j.neuroimage.2015.10.053.
13. Schilling KG, Yeh FC, Nath V, et al. A fiber coherence index for quality control of B-table orientation in diffusion MRI scans. *Magn Reson Imaging* 2019; 58: 82-89. 20190122. DOI: 10.1016/j.mri.2019.01.018.
14. Yeh F-C. Population-based tract-to-region connectome of the human brain and its hierarchical topology. *Nature Communications* 2022; 13: 4933. DOI: 10.1038/s41467-022-32595-4.
