# Supplementary material for: Identifying the white matter pathways involved in multiple sclerosis-related tremor using diffusion tensor imaging
Source: Mult Scler J Exp Transl Clin. 2023 Nov 8;9(4):20552173231208271. doi: 10.1177/20552173231208271 (PMC10631316; doi:10.1177/20552173231208271)
Supplement: sj-pdf-2-mso-10.1177_20552173231208271 - Supplemental material for Identifying the white matter pathways involved in multiple sclerosis-related tremor using diffusion tensor imaging [file sj-pdf-2-mso-10.1177_20552173231208271.pdf]

MS Journal Appendix for MRI methodology

Hardware	
Field strength	3.0 T
Manufacturer	Philips
Model	Ingenia
Coil type<br>(e.g. head, surface)	Head
Number of coil channels	15

Acquisition sequence		
Type<br>(e.g. FLAIR, DIR, DTI, fMRI)	DTI	
Acquisition time	n/a	
Orientation	axial	
Alignment<br>(e.g. anterior commissure/poster commissure line)		
Voxel size	$1 \times 1 \times 3$ mm^3	
TR	7100 ms	
TE	65 ms	
TI		
Flip angle		
NEX		
Field of view	256×256 mm^2	
Matrix size		
Parallel imaging	✓ Yes	No
If used, parallel imaging method:<br>(e.g. SENSE, GRAPPA)	SENSE	
Cardiac gating	Yes	No
If used, cardiac gating method:<br>(e.g. PPU or ECG)	n/a	
Contrast enhancement	Yes	✓ No

Acquisition sequence	
If used, provide name of contrast agent, dose and timing of scan post-contrast administration	n/a
Other parameters:	Single-shot spin-echo diffusion-sensitized echo-planar imaging sequence with a balanced Icosa21 tensor encoding scheme.<br>• b-factor = 1,000 s mm^{-2}<br>• 21 directions<br>,

Image analysis methods and outputs	
Lesions	
Type<br>(e.g. Gd-enhancing, T2-hyperintense, T1-hypointense)	n/a
Analysis method	
Analysis software	
Output measure<br>(e.g. count or volume [ml])	
Tissue volumes	
Type<br>(e.g. whole brain, grey matter, white matter, spinal cord)	n/a
Analysis method	
Analysis software	
Output measure<br>(e.g. absolute tissue volume in ml, tissue volume as a fraction of intracranial volume, percentage change in tissue volumes)	
Tissue measures (e.g. MTR, DTI, T1-RT, T2-RT, T2*, T2', ^{1}H-MRS, perfusion, Na)	
Type<br>(e.g. whole brain, grey matter, white matter, spinal cord, normal-appearing grey matter or white matter)	White matter pathways
Analysis method	ROI diffusivity measures, TBSS and Tractography
Analysis software	DTI Studio, MRICloud, FSL, and DSI Studio
Output measure	FA, MD, AD & RD
Other MRI measures (e.g. functional MRI)	
Type<br>(e.g. whole brain, grey matter, white matter, spinal cord, normal-appearing grey matter or white matter)	
Analysis method	
Analysis software	
Output measure	

Other analysis details:
